# Surgical management of single-level thoracolumbar vertebral body segmentation and formation failure causing progressive thoracolumbar myelopathy in three adult large-breed dogs

**DOI:** 10.3389/fvets.2024.1504477

**Published:** 2025-01-07

**Authors:** Francisca Couto, Joana Tabanez, Jeremy Rose, Colin Driver

**Affiliations:** Lumbry Park Veterinary Specialists, Hampshire, United Kingdom

**Keywords:** vertebral stabilization, veterinary spinal surgery, thoracic malformations, corpectomy, congenital diseases vertebral stabilization, thoracicolumbar vertebral malformations, congenital diseases

## Abstract

**Objective:**

This study aimed to evaluate the medium-term outcome following spinal cord decompression and instrumented fixation of single-level congenital thoracolumbar vertebral malformations, characterized by combined failures of segmentation and formation, causing thoracolumbar myelopathy in three large-breed dogs.

**Study design:**

This was a retrospective clinical study.

**Animals:**

The animals involved in the study were three large-breed dogs.

**Methods:**

Electronic patient records were retrospectively reviewed for adult large-breed dogs (>1 year) (>25 kg) with thoracolumbar myelopathy and a radiologic diagnosis of spinal cord compression associated with thoracolumbar vertebral malformation. The examination, diagnostic imaging, surgical management, and outcomes are described. The medium-term outcome was determined based on the neurological examination and follow-up imaging studies conducted up to 12 months post-operation.

**Results:**

Three large-breed dogs were identified, presenting with progressive, non-painful T3–L3 spinal cord segment disease. Diagnosis was made using MRI and CT, which revealed single-level complex congenital vertebral malformation with combined failures of segmentation and formation in the T8–L1 region. Surgical management consisted of ventral cord decompression by bilateral mini-hemilaminectomy and partial corpectomy and vertebral fixation. Temporary postoperative neurological deterioration was observed in two cases. Follow-up was conducted at 6 weeks (examination) and 3 (examination), 6, and 12 months (examination and CT) postoperatively, and improved neurological function was confirmed, with all cases being ambulatory with persistent, mild paraparesis.

**Conclusion:**

This retrospective study demonstrates the successful medium-term outcome following surgical management of complex thoracolumbar vertebral malformations in large-breed dogs.

## 1 Introduction

Congenital anomalies of the vertebral column are commonly identified in the thoracic spine of small-breed dogs, with French Bulldogs, English Bulldogs, Pug dogs, and Boston Terriers being overrepresented (1–5). These anomalies are less often observed in large-breed dogs, with limited case reports in puppies (2). Although the appropriate terminology has been debated, congenital thoracic vertebral malformations (CTVM) can be classified by failure of vertebral body segmentation (such as block vertebrae) and/or formation (such as hemivertebrae, wedge-shaped vertebrae, and “butterfly” vertebrae) (6, 7). This condition may predispose to abnormal angulation of the vertebral column (kyphosis, lordosis, or scoliosis), vertebral instability, canal stenosis, and spinal cord compression. Some deformations are common incidental findings in ‘at-risk’ breeds and are not commonly associated with clinical signs; others can develop neurological dysfunction with progressive, non-painful, pelvic limb ataxia, paraparesis, and incontinence. In these cases, non-surgical treatment is expected to result in a poor outcome (8). Different surgical techniques have been described with the aim of relieving spinal cord compression and stabilising the abnormal region (9, 10). In large-breed puppies, only two cases have been reported with a successful outcome following surgical treatment (1, 2). To the best of our knowledge, this is the first retrospective case series that evaluates medium-term outcomes following decompression fixation of complex thoracolumbar vertebral malformations, characterized by combined failures of segmentation and formation, in three adult large-breed dogs.

## 2 Materials and methods

### 2.1 Animals

Electronic patient records were retrospectively reviewed for adult (>1 year) large-breed dogs (>25 kg) with a radiologic diagnosis of spinal cord compression associated with thoracolumbar vertebral malformation. Three young adult (2–4 years old), intact female large-breed dogs (Rottweiler, Bloodhound, and Irish Water Spaniel) were identified (Table 1). All presented with a chronic, several-week history of progressive gait abnormalities, without apparent signs of discomfort. Cases 1 and 3 were ambulatory paraparetic with asymmetric pelvic limb ataxia (worse on the right and left, respectively) and abnormal pelvic limb postural reactions. Case 2 was non-ambulatory paraparetic with absent pelvic limb postural reactions and a cutaneous trunci cutoff at the level of T10. All three dogs were bright, alert, and responsive on initial examination. Segmental spinal reflexes and cranial nerve examination were normal. The remainder of the physical examination was unremarkable. In all dogs, complete blood count and serum biochemical profile were within normal limits. All cases had a T3–L3 spinal cord segment neuroanatomic localization. Given the progressive and non-painful history, congenital malformation, infectious/inflammatory processes, degenerative conditions, and neoplasia (e.g., intramedullary tumors) were considered most likely.

**Table 1:** Signalment and clinical summary.

Signalment	Onset	Neurological examination	Neuroanatomical localization
Dog: case 1 2 years and 9 months Rottweiler Female intact 35 kg	4–6 weeks	Ambulatory paraparesis with pelvic limb ataxia (worse on the right). Reduced paw placement and hopping on both pelvic limbs (worse on the right).	T3–L3 spinal cord segments
Dog: case 2 4 years and 5 months Bloodhound Female intact 30 kg	Since being born	Non-ambulatory paraparesis. Absent paw placement on both pelvic limbs. Cutaneous trunci around T10.	T3–L3 spinal cord segments
Dog: case 3 4 years Irish Water Spaniel Female intact 20 kg	Since 12 weeks of age	Ambulatory paraparesis with pelvic limb ataxia (worse on the left). Reduced paw placement and hopping on both pelvic limbs (worse on the left). Cutaneous trunci present around the thoracolumbar junction.	T3–L3 spinal cord segments

### 2.2 Preoperative imaging

Diagnostic imaging, including magnetic resonance imaging (MRI) and computed tomography (CT), was reviewed by at least one board-certified neurologist. MRI was acquired under general anesthesia using a 1.5 Tesla scanner (Siemens Symphony Tim System). Standard spin-echo T2-weighted (T2W) sagittal, transverse, and dorsal short tau inversion recovery (STIR) sequences were obtained through the thoracolumbar spine. CT images were obtained either under sedation or general anesthesia using a 160-slice scanner (Aquilion PRIME Toshiba, Canon Medical Systems USA, Inc., United States) with a 0.5-mm slice thickness in the transverse plane and a 1-mm reconstructed slice thickness in the sagittal and dorsal planes. CT images were used to evaluate the osseous structure for anomalous or traumatic lesions for surgical planning and postoperative follow-up. The images were evaluated using multi-planar reconstruction in a bone window using commercially available DICOM imaging software (OsiriX)^1^. ROI tools were used for linear measurements.

**Figure 1:**
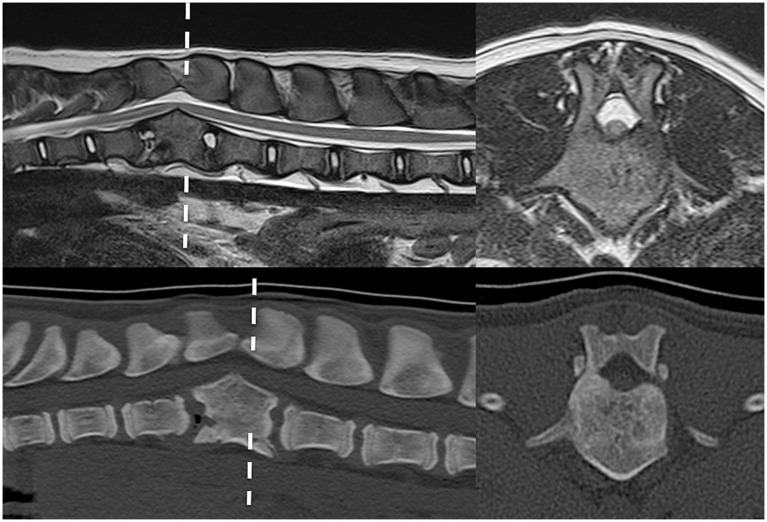
Preoperative imaging of case 1. T2W MRI (top row) and CT (bottom row) in sagittal (left) and transverse (right) planes at the level of incompletely formed and segmented T13 and L1 (dashed line represents transverse level).

**Figure 2:**
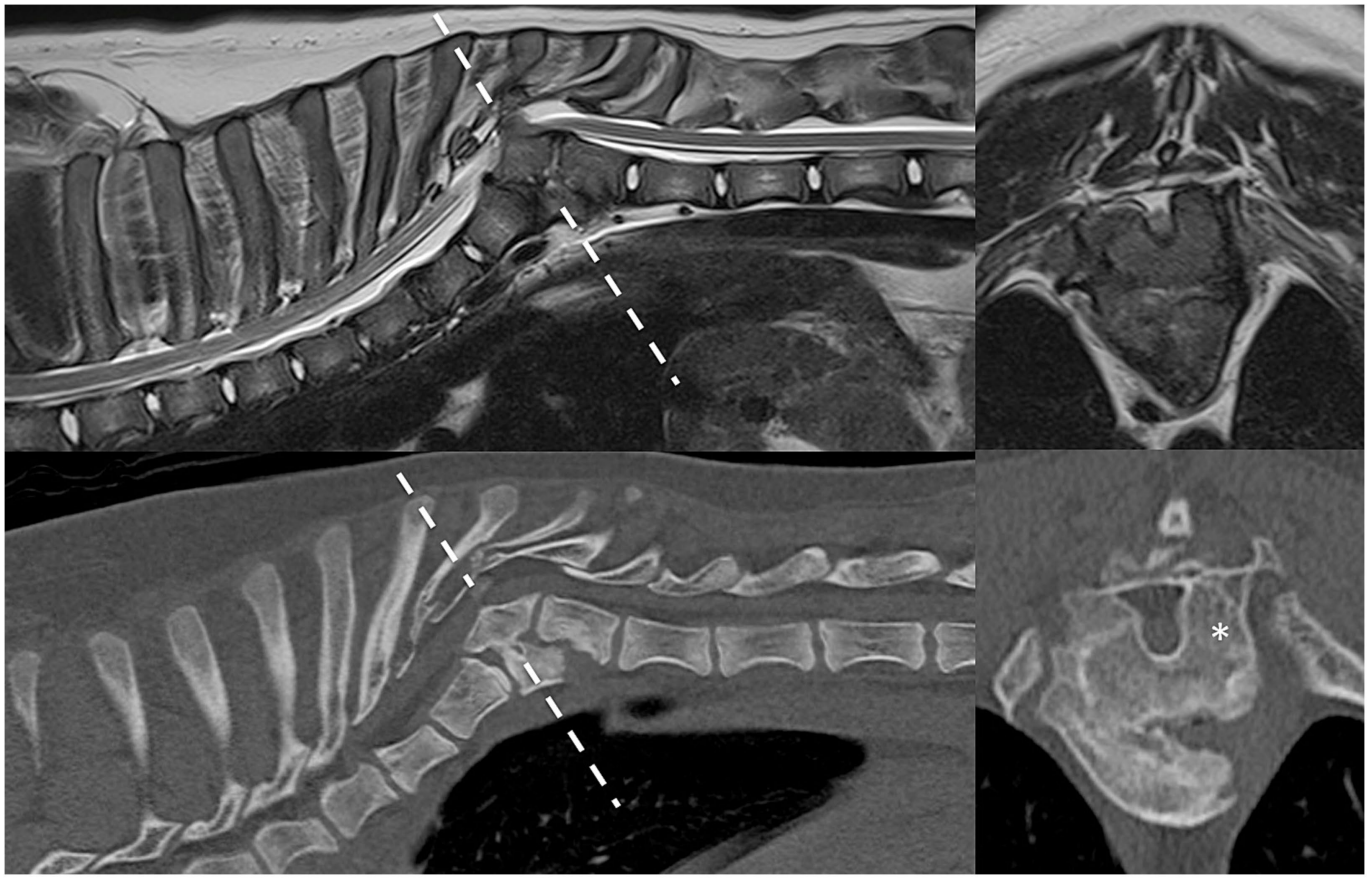
Preoperative imaging of case 2. T2W MRI (top row) and CT (bottom row) in sagittal (left) and transverse (right) planes at the level of incompletely formed and segmented T8 and T9 (dashed line represents transverse level). Asterisk = malformed left pedicle of T9 removed during surgery.

**Figure 3:**
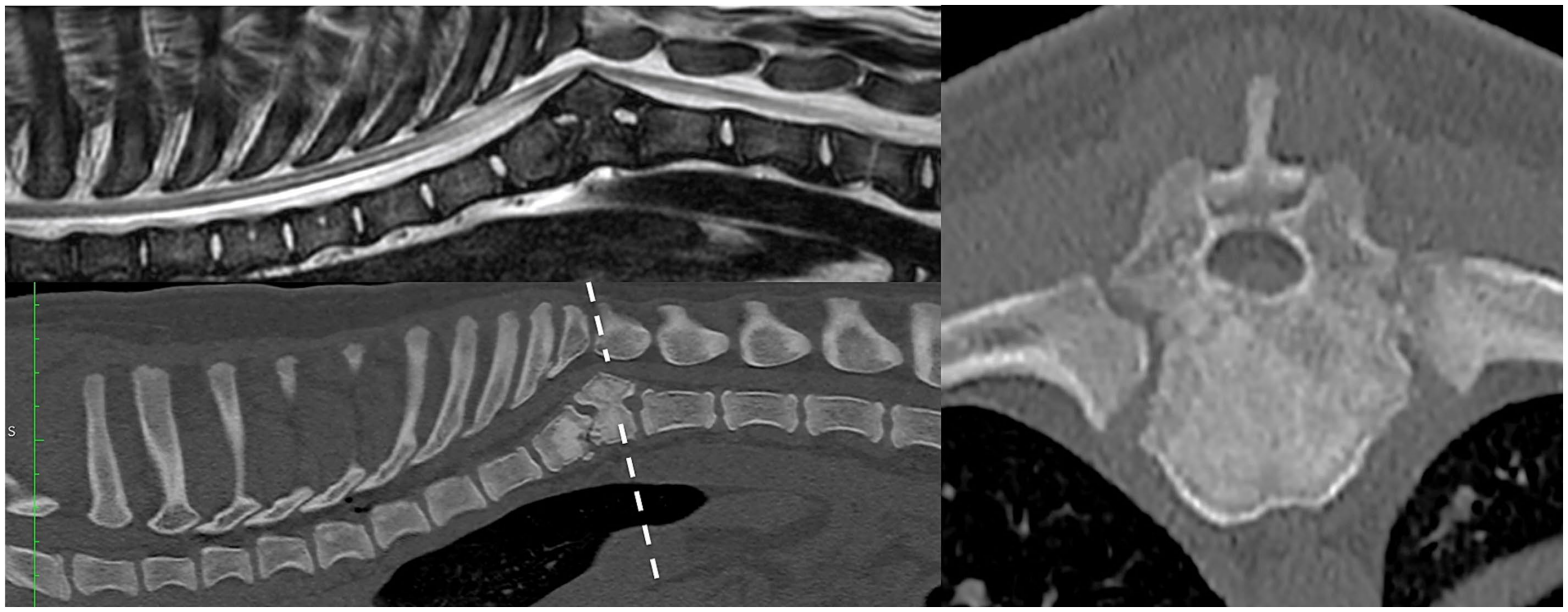
Preoperative imaging of case 3. T2W MRI (top row) and CT (bottom and right) in sagittal (left) and transverse (right) planes at the level of incompletely formed and segmented T10 and T11 (dashed line represents transverse level).

In case 1 (Figure 1), the T13 and L1 vertebral bodies were abnormally shaped and were non-segmented but had separate vertebral arches (pedicles, dorsal lamina, and spinous process). This caused a severe focal kyphotic deformity with secondary vertebral canal stenosis and ventral spinal cord compression. On T2W images in transverse view, within the cranial segment, the spinal cord diameter measured 5 × 5 mm, whereas at the apex of the malformation, it measured 4.5 × 3.7 mm. In addition, there were adjacent intervertebral disc degeneration and vertebral endplate sclerosis at T12. Case 2 had similar incomplete formation and segmentation of the T8 and T9 vertebral bodies (Figure 2), again causing significant kyphoscoliosis and spinal cord compression. Cranially, the spinal cord diameter was 5 mm, progressively decreasing to 1.8 mm and 3.5 mm above T8 and T9, respectively. In case 3 (Figure 3), there was incomplete ventral formation and segmentation of the T10 and T11 vertebral bodies. This formed one “mushroom-shaped” vertebral body, with adjacent intervertebral disc degeneration and protrusion (T9–T10 and T11–T12). In all cases, there was an increase in T2-weighted signal intensity within the spinal cord at the apex of the deformity, corresponding to the greatest degree of spinal cord compression, presumably reflecting oedema, inflammation, and/or gliosis.

### 2.3 Surgical procedures

All three cases were premedicated with methadone (0.2–0.3 mg/kg) and dexmedetomidine (3–5 mcg/kg), administered intravenously or intramuscularly. General anesthesia was then induced with propofol (4–6 mg/kg) to effect and maintained with isoflurane. Prophylactic antibiotics were given intravenously at induction (cefuroxime 20 mg/kg) and subsequently repeated every 90 min during this procedure. Perioperative analgesia was achieved with methadone (0.1–0.2 mg/kg every 4 h) and continuous rate infusion (CRI) of ketamine (5–10 mcg/kg/min).

**Figure 4:**
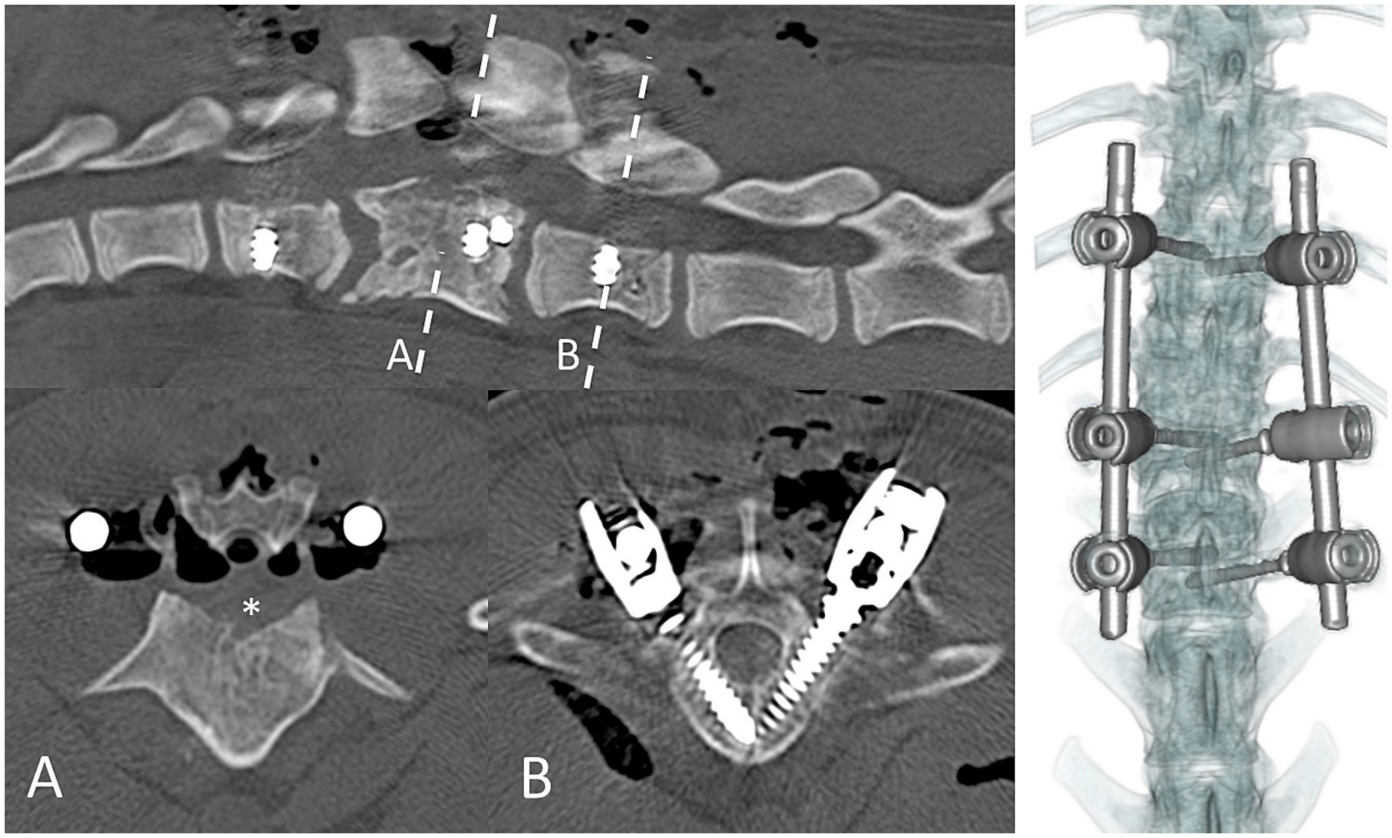
Immediate postoperative CT scan imaging of case 1. Dashed lines represent transverse levels. A = bilateral partial lateral corpectomy; asterisk denotes the region of bone removed to ventrally decompress the spinal cord. B = 4.5-mm pedicle–body screws in L2.

**Figure 5:**
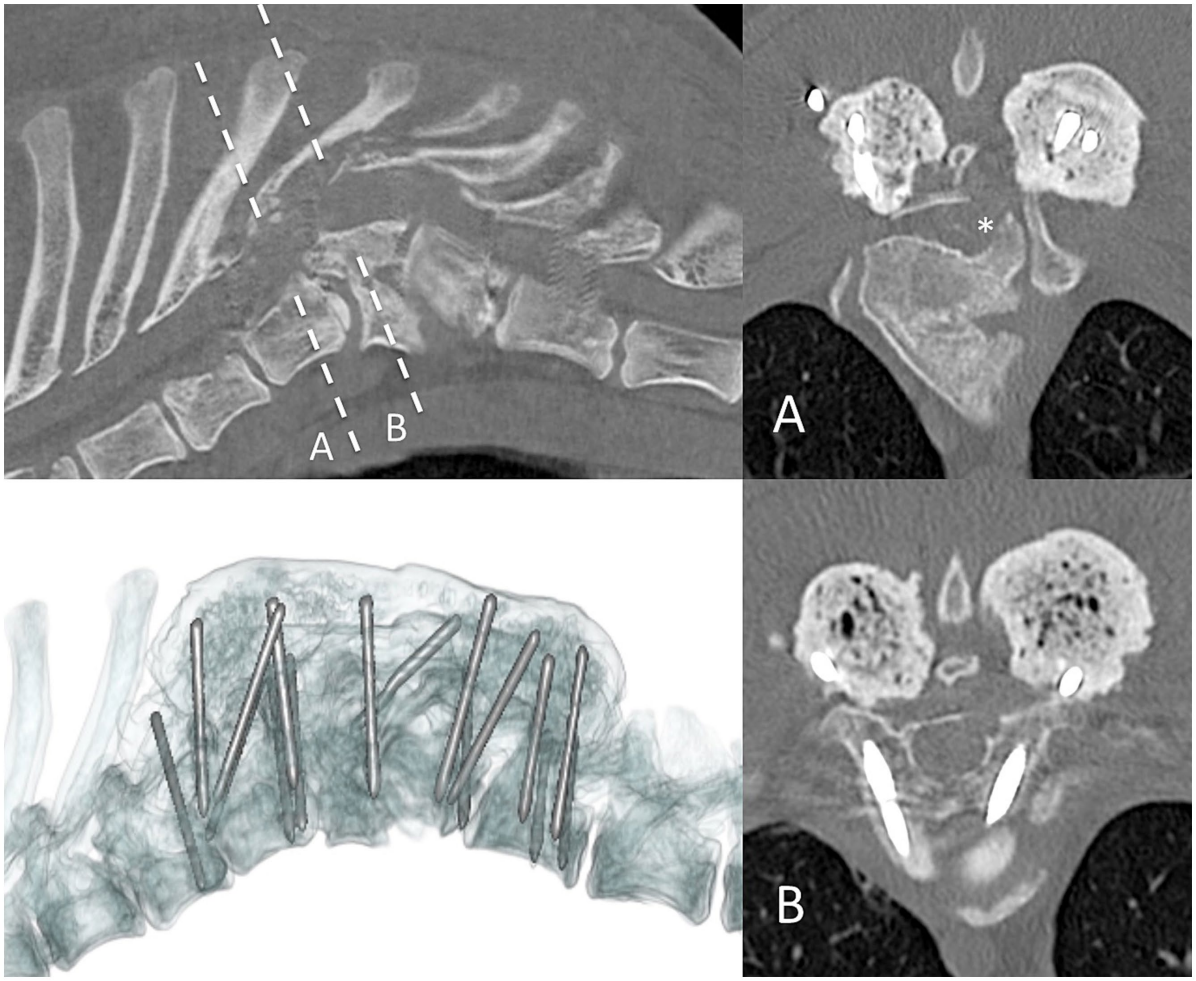
Postoperative CT scan imaging of case 2. Sagittal (top left), transverse (right column), and three-dimensional (bottom left) reconstructions. Dashed line represents the transverse level imaged. Asterisk denotes the region of bone removed from the left pedicle and dorsal aspect of the body of the T9 vertebra.

**Figure 6:**
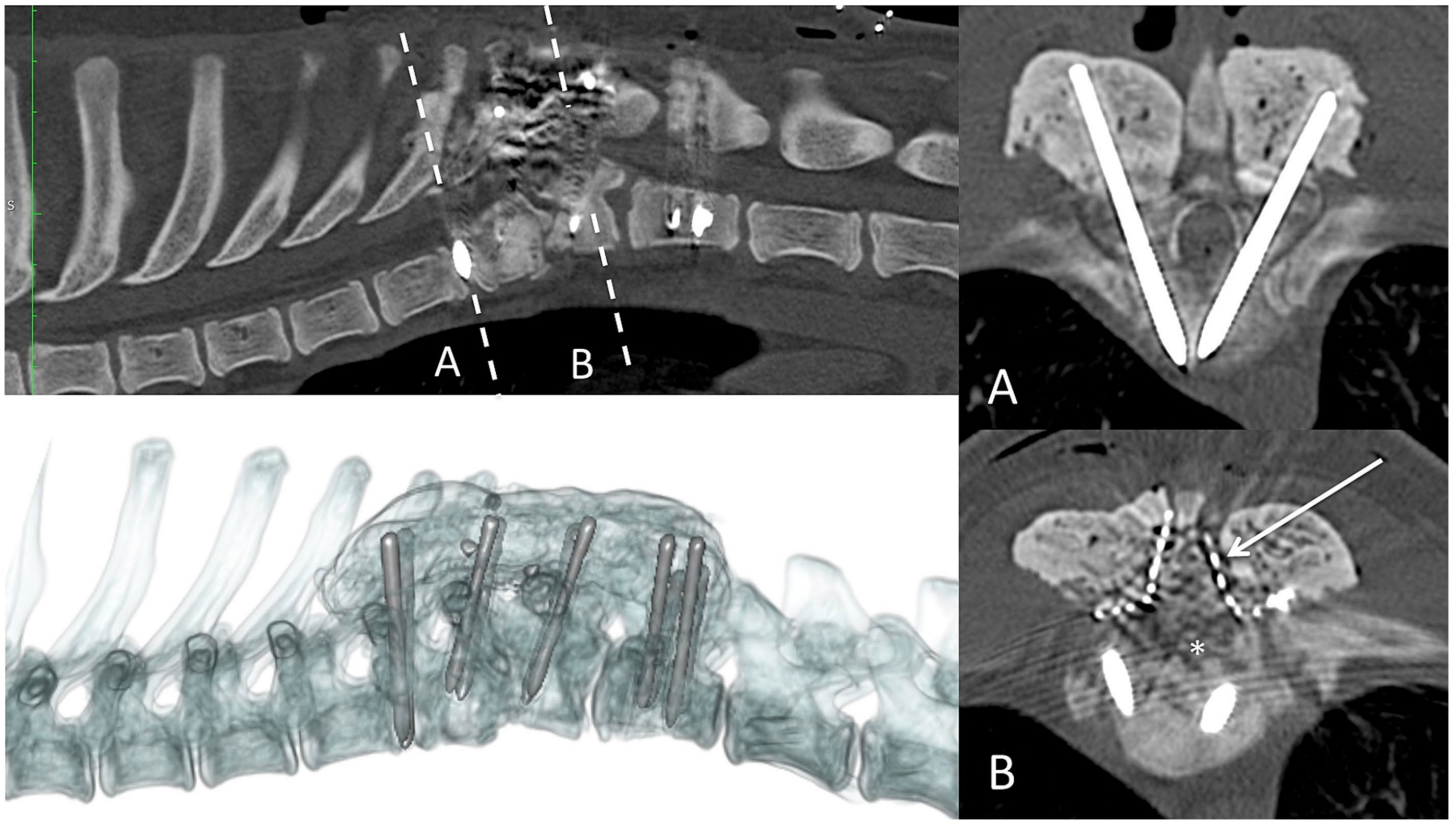
Postoperative CT scan imaging of case 3. Sagittal (top left), transverse (right column), and three-dimensional (bottom left) reconstructions. Dashed line represents the transverse level imaged. Asterisk = region of bone removed from bilateral partial lateral corpectomy. A titanium mesh laminoplasty was used to limit postoperative haematoma formation within the vertebral canal (arrow).

The dogs were positioned in sternal recumbency (as close as possible to preoperative imaging). A dorsal approach to the thoracolumbar spine was performed in all patients for a combination of ventral spinal cord decompression (via bilateral mini-hemilaminectomy and partial lateral corpectomy) and vertebral fixation with varying instrumentation. In case 1 (Figure 4), pedicle screw–rod fixation was performed using 4.0-mm diameter titanium alloy polyaxial screws (Fitzbionic Ltd., Surrey, UK) and 5.0-mm diameter interconnecting rods. In cases 2 (Figure 5) and 3, pins (2.4-mm diameter stainless steel threaded interface pins, Imex Veterinary Inc., Movora, Texas, USA) and bone cement (gentamicin-impregnated polymethylmethacrylate, PMMA, Stryker, Michigan, USA) were used. Vertebral pedicle and body implants were placed in a bi-cortical fashion using a free-hand technique along trajectories and depths determined from preoperative planning CT. In case 3 (Figure 6), a bilateral laminoplasty was subsequently performed using a contoured titanium alloy mesh (Veterinary Instrumentation, Sheffield, UK) anchored to a rib head with 2.0-mm self-tapping self-drilling screws and bone cement. A postoperative CT scan, taken immediately after the surgery, showed satisfactory ventral spinal cord decompression. In addition, implant placement was considered satisfactory because there was no evidence of vertebral canal breaches at any level, and the implants purchased satisfactory volumes of the vertebral pedicle and/or body according to the preoperative *in silico* plans.

Postoperatively, all dogs were prescribed intravenous analgesia with methadone (0.1–0.2 mg/kg every 4–6 h) and continuous rate infusion (CRI) of ketamine (2–5 mcg/kg/min), according to pain scores. In addition, they were treated with oral gabapentin (10 mg/kg twice daily), paracetamol (10 mg/kg three times daily), and non-steroidal anti-inflammatory drugs such as meloxicam (0.1 mg/kg) or robenacoxib (1–2 mg/kg), once daily.

## 3 Results

### 3.1 Clinical outcome and follow-up

In two dogs (cases 1 and 2), transient neurological deterioration was evident, with both dogs suffering significant diminishment of voluntary motor function but with intact deep pain sensation. In addition, the dog in case 2 revealed spinal hyperesthesia associated with the incision site. In both cases, the following day, revision surgery revealed large compressive epidural hematomas, which were aspirated. The duration of hospitalization was 19 and 22 days for cases 1 and 2, respectively. Both dogs were discharged as non-ambulatory paraparetic but were ambulatory at the 6-week follow-up. Case 3 did not deteriorate postoperatively and was discharged after 9 days of hospitalization with ambulatory paraparesis. Preoperative and postoperative urinary and fecal incontinence was not perceived in these patients. Postoperative recommendations included restricted rest, with a gradual increase in the length of controlled lead walks, and physiotherapy by a certified canine rehabilitation therapist. Follow-up by a neurologist included repeated neurological examination at 6 weeks, and at 3, 6, and 12 months after surgery. Improvement was observed in all three dogs, with persistent but improved ambulatory paraparesis, fewer mistakes in paw placement, and better coordination. Two cases underwent follow-up CT scans at 6 and 12 months, which revealed unchanged vertebral canal volume and implants from the postoperative period.

## 4 Discussion

Congenital thoracic vertebral malformations are believed to be caused by the failure of vertebral ossification centers to form, fuse properly, or both, during embryonic or fetal development (4, 11). A variety of thoracic malformations have been reported in small-breed dogs with kyphosis, including wedge-shaped thoracic “hemivertebrae” and “butterfly” vertebrae, which are commonly observed in the mid to caudal thoracic vertebral region, between T6 and T9. Despite being rarely reported in large-breed dogs, congenital thoracic vertebral malformations in small animals can result in progressive kyphotic deformation of the vertebral column, with spinal cord compression and vertebral canal stenosis typically manifesting in young dogs (4, 9, 10, 12–14). In this retrospective study, a complex combined failure of vertebral formation and segmentation between T8 and L1 was associated with focal but marked kyphosis of the vertebral column at a single level in adult large-breed dogs.

Progression of clinical signs and poor outcomes have been reported in multiple studies after non-surgical treatment (8, 9, 14). Surgical techniques include fixation with or without spinal cord decompression and decompression without fixation (2, 9). Different surgical techniques for spinal decompression have been described (including dorsal laminectomy, hemilaminectomy, and/or corpectomy), and several strategies for spinal fixation (13). Two previous reports in large-breed dogs have demonstrated successful outcomes with surgical spinal decompression and fixation (1, 2). One report described a partial ventral corpectomy and fusion with pins and cement (1). Another described unilateral hemilaminectomy and lateral corpectomy, followed by bilateral stabilization with pins and PMMA bars (2). Although spinal fixation is not always considered necessary, the decision to proceed with it should take into consideration multiple factors, including the extent of the surgical technique used for spinal cord decompression (such as bilateral hemilaminectomy over multiple vertebrae, partial corpectomy, radical dorsal laminectomy, or hemilaminectomy with corpectomy), the three-compartment concept of spinal stability (i.e., if two or more compartments are compromised, the vertebral column should be considered unstable), disease chronicity, and imaging findings suggestive of instability (13, 15). In the current case series, given the ventral spinal cord compression, decompression with a bilateral mini-hemilaminectomy followed by a partial lateral corpectomy was considered necessary. In addition, different strategies for spinal fixation have been reported, and the most commonly used instrumentation includes screws and plates, screws/pins, and PMMA (5, 13, 15). Furthermore, surgical experience, anatomical considerations, and the type of spinal fixation required help with the implant selection (15). Polyaxial pedicle screws and rods were used in case 1 due to implant availability and ease of application in the thoracolumbar region. Cases 2 and 3 were managed with threaded pins and PMMA due to appropriate implant sizing and ease of use.

Similar to previous reports in large-breed dogs treated surgically (1, 2), two of the three dogs in the present study demonstrated an immediate postoperative neurological deterioration. Postoperative CT scan and revision surgery confirmed the presence of epidural hematoma formation. Although considered unlikely, we cannot rule out the possibility of an underlying coagulopathy being the cause of the hematoma in cases 1 and 2. Further tests, such as a coagulation profile, were not performed in all three cases. Other complications, following surgical treatment, have been previously reported, such as iatrogenic injury to the spinal cord, nerve roots, intervertebral discs, and vascular structures, as well as invasion of the thoracic cavity, abnormal positioning of the implant, implant failure, or infection (9, 13, 15). Given the deterioration in both cases 1 and 2, in case 3, bilateral laminoplasty was performed to help limit the risk of postoperative spinal cord compression related to hematoma and dependent oedema formation, which was thought to be more of a risk in large-breed dogs due to the depth of the surgical wound and extent of muscle dissection. It is unclear whether this attempt was successful, but it may be beneficial in future cases.

Although all dogs in the present study gradually improved at 6 weeks, and at 3, 6, and 12 months after surgery, they remained with a mild residual ataxic gait. This incomplete recovery has also been reported in previous studies and may be associated with the chronicity and irreversible damage to the spinal cord before surgery (9).

The limitations of this study include its retrospective nature and the small study population. A recent study discussing a congenital malformation in a large-breed dog treated with corpectomy and stabilization, reported a good prognosis with a long-term outcome of 34 months. This patient displayed a persistent, mild paraparesis (2). In addition, another study in brachycephalic dogs, investigating surgically managed malformations, reported chronic complications in 58% of the cases. A long-term follow-up was documented between 2006 and 2020 (14). This retrospective case series suggests that successful medium-term outcomes are possible in the surgical management of large-breed dogs with complex thoracolumbar vertebral malformations suffering from myelopathy. However, long-term evaluation should be considered in order to further evaluate potential chronic complications associated with surgery.

## Data Availability

The raw data supporting the conclusions of this article will be made available by the authors, without undue reservation.

## Footnotes

^1^ www.osirix-viewer.com
